# *ERG* Induces Epigenetic Activation of Tudor Domain-Containing Protein 1 (*TDRD1*) in *ERG* Rearrangement-Positive Prostate Cancer

**DOI:** 10.1371/journal.pone.0059976

**Published:** 2013-03-29

**Authors:** Lukasz A. Kacprzyk, Mark Laible, Tatjana Andrasiuk, Jan C. Brase, Stefan T. Börno, Maria Fälth, Ruprecht Kuner, Hans Lehrach, Michal R. Schweiger, Holger Sültmann

**Affiliations:** 1 Unit Cancer Genome Research, Division of Molecular Genetics, National Center for Tumor Diseases (NCT) and German Cancer Research Center (DKFZ), Heidelberg, Germany; 2 Department of Vertebrate Genomics, Max Planck Institute for Molecular Genetics, Berlin, Germany; 3 Department of Biology, Chemistry and Pharmacy, Free University Berlin, Berlin, Germany; University of Texas Health Science Center, United States of America

## Abstract

**Background:**

Overexpression of ERG transcription factor due to genomic *ERG*-rearrangements defines a separate molecular subtype of prostate tumors. One of the consequences of ERG accumulation is modulation of the cell’s gene expression profile. Tudor domain-containing protein 1 gene (*TDRD1*) was reported to be differentially expressed between *TMPRSS2:ERG*-negative and *TMPRSS2:ERG*-positive prostate cancer. The aim of our study was to provide a mechanistic explanation for the transcriptional activation of *TDRD1* in ERG rearrangement-positive prostate tumors.

**Methodology/Principal Findings:**

Gene expression measurements by real-time quantitative PCR revealed a remarkable co-expression of *TDRD1* and *ERG* (r^2^ = 0.77) but not *ETV1* (r^2^<0.01) in human prostate cancer *in vivo*. DNA methylation analysis by MeDIP-Seq and bisulfite sequencing showed that *TDRD1* expression is inversely correlated with DNA methylation at the *TDRD1* promoter *in vitro* and *in vivo* (ρ = −0.57). Accordingly, demethylation of the *TDRD1* promoter in *TMPRSS2:ERG*-negative prostate cancer cells by DNA methyltransferase inhibitors resulted in *TDRD1* induction. By manipulation of *ERG* dosage through gene silencing and forced expression we show that ERG governs loss of DNA methylation at the *TDRD1* promoter-associated CpG island, leading to *TDRD1* overexpression.

**Conclusions/Significance:**

We demonstrate that ERG is capable of disrupting a tissue-specific DNA methylation pattern at the *TDRD1* promoter. As a result, *TDRD1* becomes transcriptionally activated in *TMPRSS2:ERG*-positive prostate cancer. Given the prevalence of ERG fusions, *TDRD1* overexpression is a common alteration in human prostate cancer which may be exploited for diagnostic or therapeutic procedures.

## Introduction

Approximately half of human prostate cancer cases identified by PSA-screening harbor genomic rearrangements in which androgen-responsive regulatory elements are juxtaposed to genes coding for transcription factors of the ETS family [1]–[3]. As a result, ETS genes become coupled to androgen receptor (AR) signaling and are overexpressed in fusion-positive prostate tumors [4]–[6]. The most prevalent of these genomic rearrangements, the *TMPRSS2*:*ERG* gene fusion, leads to a strong overexpression of the ERG transcription factor which is otherwise absent in cells of the prostate epithelium [7]; under physiological conditions *ERG* displays a tissue-restricted expression pattern and is transcribed in the hematopoietic linage [8] and endothelial cells [9]. The question of how ERG accumulation influences the biology of prostate cancer cells *in vitro* and *in vivo* has gained a significant interest. Until now, ERG was suggested to modulate the phenotype of prostate cancer cells by a wide range of processes, including: disruption of AR signaling [10], activation of c-myc signaling [11] and estrogen receptor network [12], activation of the Wnt pathway and induction of epithelial-to-mesenchymal transition [13], promotion of cell invasion [14], physical interaction with PARP1 [15] and activation of TGF-β/BMP signaling [16]. Tumors harboring the ERG fusion were also found to be enriched for loss of the *PTEN* tumor suppressor [17], [18]. Accordingly, in mouse models of prostate cancer ERG was shown to cooperate with PI3K pathway to drive carcinogenesis [19], [20]. Accumulation of ERG was also found to be associated with an altered DNA methylation pattern in prostate cancer cells [10], [21], [22]. Analysis of the prostate cancer transcriptome performed by us and others demonstrated that tumors harboring the *TMPRSS2*:*ERG* fusion share a unique gene expression profile which significantly differs from profiles of benign prostate tissue and malignant tumors lacking the fusion [1], [10], [12], [13], [16], [23]. Specifically, tumors overexpressing ERG are characterized by transcriptional modulation of genes involved in the Wnt and TGF-β/BMP pathways [16], β-estradiol network [12], [23] and NF-κB pathway [24].

Among genes deregulated in *ERG*-rearranged prostate cancer, at least two independent studies identified Tudor domain-containing protein 1 (*TDRD1)* as the most differentially expressed gene between *ERG* rearrangement-positive and -negative prostate cancer, apart from *ERG* itself [16], [23]. Similarly to *ERG*, *TDRD1* is not transcribed in normal prostate epithelium [25], [26]. *TDRD1* has been initially identified as a cancer/testis antigen, i.e. a gene which is expressed in the testis and cancer, but silent in adult somatic tissues [26]. Its mouse ortholog, *Tdrd1*, is expressed during spermatogenesis where it acts in the conserved piRNA pathway to repress the activity of LINE1 retrotransposons by methylation [27]. A recent study in zebrafish suggested that Tdrd1 acts as a molecular scaffold for Piwi proteins, piRNAs and piRNA targets [28]. In both mouse and zebrafish, Tdrd1 is required for a correct function of the piRNA pathway and *Tdrd1* knockout in mouse results in a defective spermatogenesis [28], [29].

Here, we report that *ERG* and *TDRD1* are co-expressed in human prostate cancers and we provide a mechanistic explanation for the observed co-expression. We demonstrate that ERG activates *TDRD1* transcription by inducing loss of DNA methylation at the *TDRD1* promoter-associated CpG island. We propose that this epigenetic consequence of the *TMPRSS2:ERG* fusion represents a novel mechanism which may explain part of the transcriptional modulation induced by ERG in human prostate cancer.

## Materials and Methods

### Ethics Statement

Prostate tissue samples were obtained from the University Medical Center Hamburg Eppendorf. Approval for the study was obtained from the local ethics committee and all patients agreed to additional tissue sampling for scientific purposes.

### Prostate Tissue Samples, Genome-wide Expression Profiling and Methylation Analysis

Details of human samples collection, extraction of RNA, conversion to cDNA and genome-wide expression profiling are described elsewhere [16]. DNA extraction and genome-wide methylation analysis by MeDIP-Seq are described elsewhere [22]. The data from genome-wide expression profiling and genome-wide methylation analysis are publicly available in the Gene Expression Omnibus database (accession numbers GSE29079 and GSE35342). *TMPRSS2:ERG* fusion status was determined by PCR using previously described primers [30] and by qPCR [16]. Samples, for which both mRNA expression and DNA methylation data were available, were included in the analysis.

### Cell Culture

VCaP, NCI-H660, LNCaP, DU145, PC-3, and RWPE-1 cells were obtained from ATCC (Manassas, VA, USA). BPH-1 cells were a kind gift of Doris Mayer (DKFZ, Heidelberg). K-562 and KG-1 cells were provided by Christoph Plass and Peter Krammer, respectively (DKFZ, Heidelberg). MOLT4 and CMK cells were obtained from DSMZ (Braunschweig, Germany). VCaP cells were maintained in DMEM medium (Gibco, Life Technologies, Carlsbad, CA, USA) supplemented with 10% FBS (Gibco). NCI-H660 cells were cultured in RPMI-1640 (Gibco) supplemented with 5% FBS (Gibco), 2 mM L-gluatmine, 0.005 mg/ml insulin, 0.01 mg/ml transferrin, 30 nM sodium selenite, 10 nM hydrocortisone and 10 nM beta-estradiol (all from Sigma-Aldrich, St Louis, MO, USA). LNCaP and DU145 were maintained in RPMI-1640 (Gibco) supplemented with 10% FBS. PC-3 cells were cultured in F12-K medium (ATCC) supplemented with 10% FBS. RWPE1 cells were cultured in keratinocyte serum-free medium supplemented with 0.05 mg/ml bovine pituary extract and 5 ng/ml recombinant EGF (Gibco). BPH1 cells were cultured in RPMI-1640 medium (Gibco) supplemented with 10% FBS and 20 ng/mL 5α-dihydrotestosterone (Sigma). K-562 and MOLT-4 cells were cultured in RPMI-1640 and supplemented with 10% heat-inactivated FBS, KG-1 and CMK cells were cultured in RPMI-1640 supplemented with 20% heat-inactivated FBS.

### Generation of Stable LNCaP Clones and Induction of Transgene Expression

ERG coding sequence (exons 4–11 from NM_004449.3), which corresponds to TMPRSS2:ERG fusion T1/E4 [31], was amplified from NM_004449.3-containing plasmid (Genomics and Proteomics Core Facility, DKFZ ) by PCR using primers: forward GCAGGCTCCACCATGACCGCGTCCTCCTCCAG, reverse CAAGAAAGCTGGGTCTTAGTAGTAAGTGCCCAGAT. Stably transfected LNCaP clones were generated as previously described [32]. Transgene expression was induced with 50 ng/ml doxycycline (Sigma).

### RNA Extraction and Reverse Transcription

Total RNA was isolated from exponentially growing cell lines using RNeasy Mini Kit (Qiagen, Hilden, Germany) following the manufacturer’s instruction. cDNA synthesis was performend using SuperScript III reverse transcriptase (Life Technologies) and oligo-dT primers (Sigma-Aldrich) following manufacturers’ instructions. For the measurement of LINE1-ORF2 mRNA, total RNA was treated with Turbo DNase (Life Technologies) to remove the contaminating genomic DNA. DNase-treated RNA was then purified using RNeasy MinElute Cleanup Kit (Qiagen) and subjected to reverse transcription using RevertAid H Minus First Strand cDNA Synthesis Kit (Fermentas, Burlington, Canada) and random hexamer primers.

### Quantitative RT-PCR

Gene expression levels were measured on the LightCycler 480 Real-Time PCR System (Roche, Mannheim, Germany). cDNA equivalent of 10 ng total RNA was used per well. All measurements were performed in triplicate. Taqman assays (Applied Biosystems) were run with 2x ABsolute QPCR Mix (Abgene, Thermo Fischer, Epsom, UK). Universal Probe Library (UPL) system assays (Roche) were run using 480 Probes Master (Roche). Raw Cp values were calculated by the Roche Lightcycler 480 software using the 2nd derivative maximum method. Assays and primer sequences are listed in the Table S1 together with the corresponding figure numbers. Expression levels are presented as absolute values (Cp) or as expression relative to an internal reference gene (using ΔCp method).

### Western Blotting

Exponentially-growing cells were lysed with RIPA buffer (50 mM Tris-HCl pH 8, 150 mM NaCl, 1% NP-40, 0.5% sodium deoxycholate, 0.1% SDS) supplemented with 5 mM EDTA and HALT Protease Inhibitor Cocktail (Pierce, Thermo Fisher Scientific, Rockford, IL). Protein concentrations were determined with BCA Protein Assay kit (Pierce). Unless stated otherwise, 30 µg of protein lysates were separated in 10% SDS-PAGE gels and blotted onto nitrocellulose membranes (Macherey-Nagel, Düren, Germany) and probed with the antibodies listed in the Table S1. Signals were detected using ECL substrate solution [33] and recorded with Fusion-SL 3500 WL image acquisition system (Vilber Lourmat, Marne-la-Vallée, France).

### siRNA-mediated Gene Silencing

All siRNAs used in the study were synthesized by Dharmacon (Thermo Fisher Scientific, Epsom, UK) and resuspended in the 1x siRNA Buffer (Dharmacon). Unless indicated otherwise, cells were seeded one day before transfection at the confluence of 50–70%. siRNA transfection was performed using Lipofectamine RNAi Max (Life Technologies) according to the manufacturer’s instructions. Transfection complexes were prepared in serum-free OptiMEM medium (Gibco) and added to a complete growth medium in 20∶80 v/v proportion. Final concentrations of siRNAs were 50 nM (non-targeting pool siRNA,ERG siRNA) or 25 nM (TDRD1 siRNAs). All siRNA used in the study are listed in the Table S1.

### CpG Island Definition and Bisulfite DNA Sequencing

*TDRD1*-promoter associated CpG island was defined according to the default criteria provided by UCSC Genome Browser (http://genome.ucsc.edu/). Genomic DNA was extracted from cells using QIAamp DNA blood Mini Kit (Qiagen). Sodium bisulfite conversion of DNA was performed with the EpiTect Bisulfite Kit (Qiagen) using 1µg of genomic DNA. The 525-bp DNA fragment containing the TDRD1 promoter-associated CpG-island was amplified with HotStarTaq DNA Polymerase (Qiagen) using primers listed in Table S1. The PCR product was cloned into pCR2.1 vector using TOPO TA cloning kit (Life Technologies). TOP10 chemically competent cells (Life Technologies) were transformed with the ligation product. After blue-white screening, plasmids from the colonies containing the insert were subjected to Sanger sequencing (GATC, Konstanz, Germany). Raw Sanger sequencing reads were analyzed for methylation events using the online tool BISMA [34].

### 5-aza-2′-deoxycytidine Treatment

LNCaP or VCaP cells were seeded onto poly-L-lysine (Sigma-Aldrich) coated 12-well plates at low density. As of the following day, cells were treated with vehicle (0.1% DMSO, Applichem, Darmstadt, Germany) or the indicated concentrations of 5-aza-2′-deoxycytidine (Sigma-Aldrich) for five consecutive days. Every 24 h, growth medium was replaced with a freshly-prepared medium containing either 5-aza-2′-deoxycytidine or vehicle.

### Cell Viability Assay

For the viability assay, 2.5x10^4^ VCaP cells were seeded in black bottom 96-well plates (Perkin Elmer, Waltham, MA, USA) in 80µl of the normal growth medium. After 24 h, cells were transfected with siRNAs as described above and this day was referred to as “day 1”. Cell viability was assessed with the CellTiter-Blue Cell Viability Assay (Promega Corporation, WI, USA) on days 1, 3, 5, 7 and 9 according to the manufacturer’s instructions. Fluorescence was recorded with Tecan Infinite M200 plate reader (Tecan Group Ltd, Männedorf, Switzerland).

### Statistical Data Analysis

All data were analysed using GraphPad Prism 5.04. Quantitative data are shown as mean ± SEM (standard error of the mean) calculated from all performed experiments, unless indicated otherwise. All comparisons between experimental groups were performed by Mann-Whitney-Wilcoxon test with Bonferroni correction (* P<0.05, ** P<0.01, *** P<0.001, **** P<0.0001). Spearman (*ρ*) and Pearson (r) correlation coefficients were calculated with GraphPad Prism 5.04.

## Results

### *TDRD1* is Co-expressed with *ERG* but not with *ETV1* in Human Prostate Cancer

**Figure 1:**
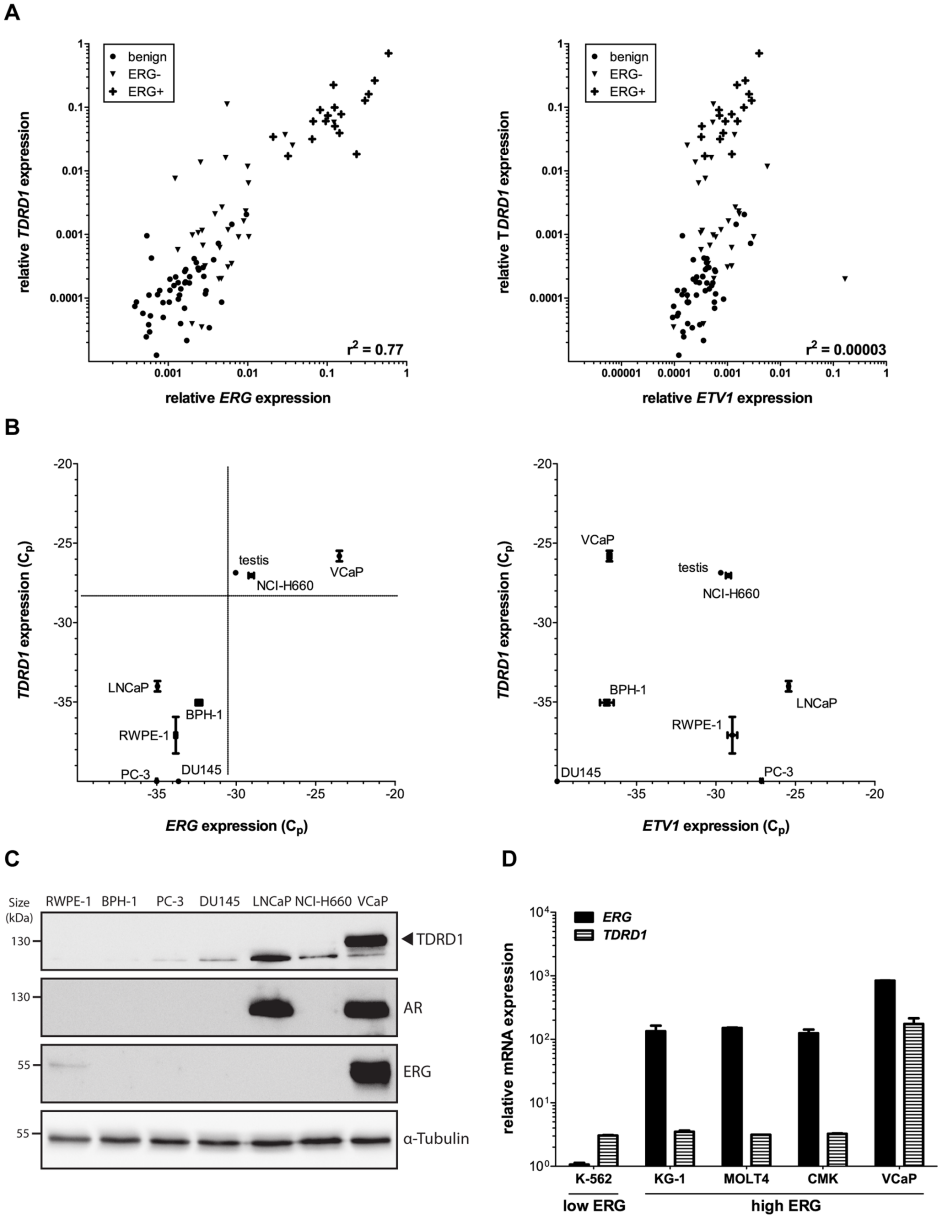
*TDRD1* is co-expressed with *ERG* but not with *ETV1* in prostate cancer. (A) Correlation analysis of mRNA levels measured by qRT-PCR in *TMPRSS2:ERG*-negative (ERG-, n = 30) and *TMPRSS2:ERG*-positive (ERG+, n = 17) prostate cancers as well as adjacent benign prostate tissue (n = 46). Pearson correlation coefficient is shown. (B) Analysis of mRNA expression in prostate cell lines by qRT-PCR. Two independent experiments were performed in triplicate. Human testis RNA was used as positive control for *TDRD1* expression. (C) Analysis of protein expression in prostate cell lines by western blotting. (D) Analysis of mRNA expression in hematopoietic cancer cell lines by qRT-PCR. Two independent experiments were performed in triplicate.

Our previous expression profiling study of human prostate cancer specimens revealed that *TDRD1* is, apart from *ERG*, the most differentially expressed gene between *TMPRSS2*:*ERG*-negative and -positive tumors [16]. We thus performed a correlation analysis on the data from 93 prostate tissue samples (46 benign, 30 *TMPRSS2:ERG*-negative, 17 *TMPRSS2:ERG*-positive prostate tumors) and found that mRNA levels of *ERG* and *TDRD1* measured by Human Exon 1.0 ST Array are remarkably correlated across all samples (r^2^ = 0.84), suggesting a mechanistic link between the two genes. In contrast, *TDRD1* was not co-expressed with *ETV1* (r^2^ = 0.05) which is an ETS transcription factor found to be sporadically rearranged in prostate cancer. To corroborate these observations, we measured *TDRD1*, *ERG* and *ETV1* mRNA levels with quantitative RT-PCR in the same set of samples. Again, *TDRD1* expression was found to correlate with *ERG* (r^2^ = 0.77), but not with *ETV1* (r^2^<0.01) expression (Fig. 1a). To provide an independent validation of our findings, we queried the Oncomine database [35] using “TDRD1” and “prostate cancer” as search terms. The analysis of two identified studies supports our observations: in the data of Grasso et al. [36] *ERG* was the topmost gene co expressed with *TDRD1*. In the data of Taylor et al. [17], *TDRD1* was found to be co expressed with *ERG* (r^2^ = 0.55) but not with *ETV1* (r^2^ = 0.02) across 149 primary prostate tumors. To explain the observed co-expression, we employed cellular models of prostate cancer, including cells representing benign (RWPE-1, BPH-1), fusion-negative (PC-3, DU145), *ERG*-rearranged (VCaP, NCI-H660) and *ETV1*-rearranged (LNCaP) prostate cancer. Gene expression measurements in prostate cell lines showed that while *TDRD1* mRNA levels were independent of *ETV1* expression, an evident association exists between *TDRD1* and *ERG* expression *in vitro* (Fig. 1b). None of the cell lines without *ERG* overexpression expressed *TDRD1*, while NCI-H660 and VCaP cell lines, both of which harbor the *TMPRSS2:ERG* fusion, expressed high levels of *TDRD1* (Fig. 1b). We then asked if the high levels of both *TDRD1* and *ERG* messenger RNA in ERG-positive prostate cells translate into considerable amounts of the respective proteins and found that VCaP cells express ERG and TDRD1 at levels detectable by western blotting (Fig. 1c). Based on these initial results, we decided to use VCaP cells as an *in vitro* model to study the mechanistic relation between *ERG* and *TDRD1* genes in *ERG-*rearranged prostate cancer.

### *TDRD1* is not co-expressed with *ERG* in Hematopoietic Cancers

Some hematopoietic cancers overexpress ERG protein [37], [38] and it is known that myeloid, T- and Bcell leukemias depend on ERG for their maintenance [39], [40]. We thus investigated *ERG* and *TDRD1* co-expression by qRT-PCR in a panel of cell lines representing various hematopoietic cancers. Although we detected high levels of *ERG* mRNA in several cancer cell lines derived from the hematopoietic lineage, the corresponding expression of *TDRD1* mRNA was approximately 50-fold lower than in VCaP cells (Fig. 1d). To extend our analysis beyond cell lines, we compared *ERG* and *TDRD1* mRNA expression in prostate tissues and in cytogenetically abnormal acute myeloid leukemia (CA-AML) measured by the same platform (Human Exon 1.0 ST Array) [41]. In contrast to our observations in prostate cancer (r^2^ = 0.84), *ERG* and *TDRD1* were not co-expressed in CA-AML (r^2^ = 0.07). *ERG* has also been reported to be overexpressed in Ewing’s sarcomas [42]–[45]. We thus analyzed the available gene expression profiling studies of Ewing’s sarcoma tumors for *TDRD1* and *ERG* expression [46], [47]. In contrast to prostate cancer, there was no co-expression of *ERG* and *TDRD1* in any of these studies (r^2^ = 0.03 and 0.02, respectively). This suggests that the co-expression of *ERG* and *TDRD1* is specific for prostate cancer.

### ERG Transcription Factor is Required to Maintain High *TDRD1* Expression in *TMPRSS2:ERG*-positive Cells

**Figure 2:**
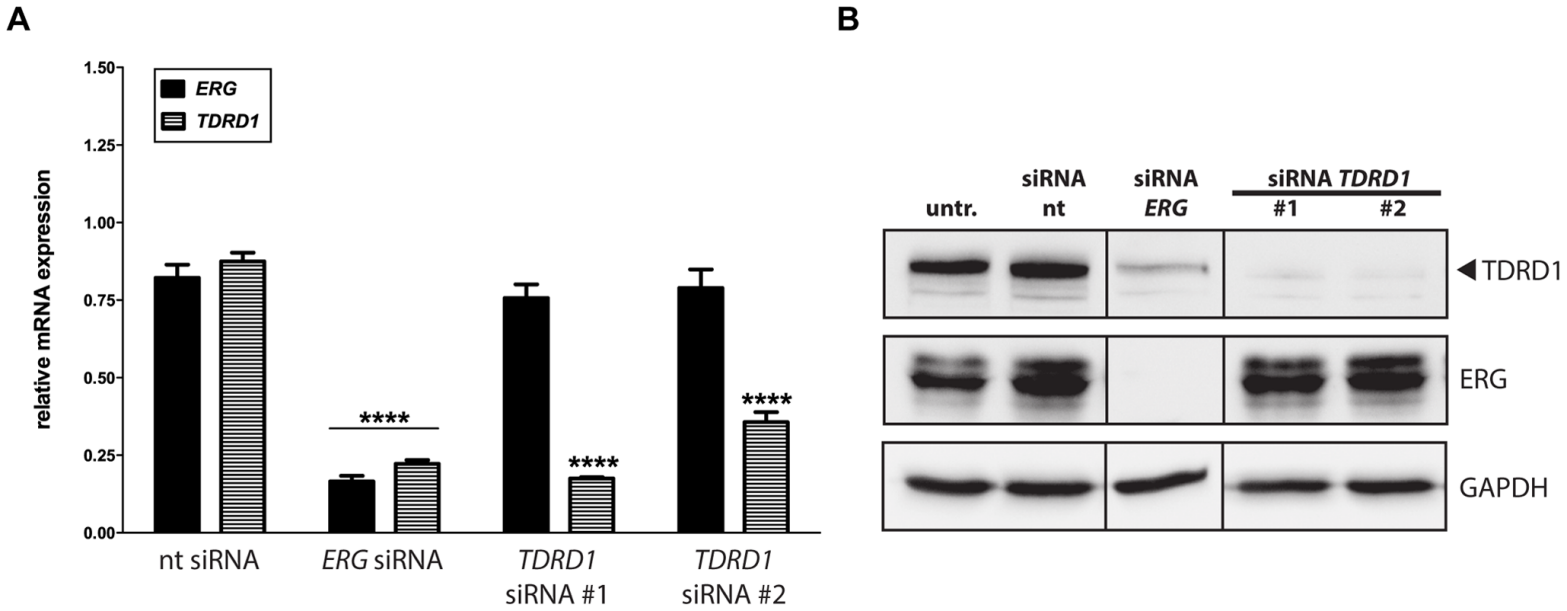
ERG transcription factor is required to maintain high *TDRD1* expression. (A) *ERG* and *TDRD1* mRNA expression levels in VCaP cells measured 72 h after gene silencing with siRNAs. Three independent experiments were performed in triplicate. (B) ERG and TDRD1 protein expression in VCaP cells 72 h after gene silencing with siRNAs.

Co-expression of two genes can be explained by, among others, regulation of one of the genes by the other or by their mutual regulation in a feed-forward loop. To test these possibilities, we depleted either ERG or TDRD1 protein in VCaP cells by RNA interference and determined mRNA and protein expression of both genes. Silencing of *ERG* with 80% efficiency resulted in 3.9-fold downregulation of *TDRD1* mRNA 72 h post-transfection (P<0.0001, Fig. 2a). In contrast, silencing of *TDRD1* did not result in any changes in *ERG* mRNA expression. Analysis of the corresponding protein levels by western blot revealed that silencing of *ERG* and *TDRD1* genes was very effective, leading to a complete depletion of both proteins from the cells (Fig. 2b). While knockdown of *ERG* caused a profound downregulation of TDRD1 protein at 72 h, no such effects on ERG were detected after silencing of the *TDRD1* gene. A similar expression pattern was also observed at 48 h after transfection (data not shown). In conclusion, a constant presence of ERG is required to maintain high expression of *TDRD1* in VCaP cells and the observed co-expression of the two genes in prostate tumors could be explained by a unidirectional activation of *TDRD1* through the ERG transcription factor.

### *TDRD1* Promoter-associated CpG Island is Hypomethylated in *TMPRSS2:ERG*-positive Prostate Tumors

**Figure 3:**
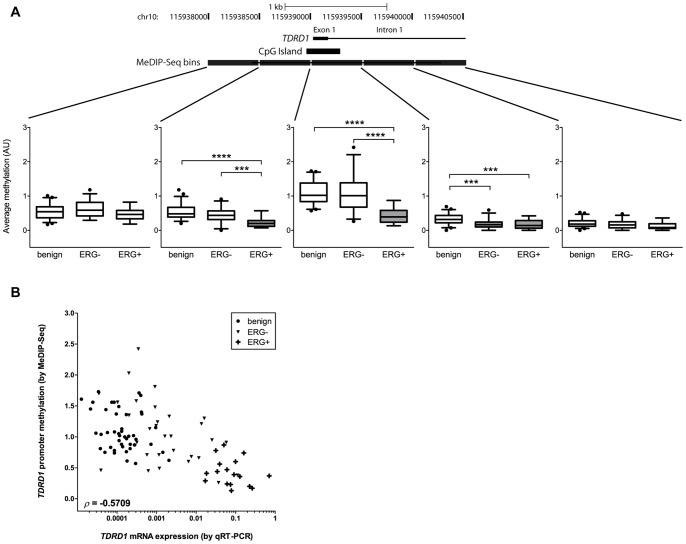
*TDRD1* promoter associated CpG island is hypomethylated in *TMPRSS2:ERG*-positive prostate cancer. (A) Analysis of *TDRD1* promoter methylation in prostate tumors by MeDIP-Seq. The values represent the average degree of DNA methylation of the 500-bp bins. (B) Correlation analysis of *TDRD1* promoter methylation and *TDRD1* mRNA expression in prostate cancer. The Spearman correlation coefficient is shown.

Expression of *TDRD1*, along with that of other germ line-specific genes, is known to be repressed in somatic tissues by epigenetic silencing [26], [48]. We therefore investigated methylation status of the *TDRD1* promoter and the associated CpG island in the context of *ERG* rearrangements. We inspected methylation of DNA around the *TDRD1* transcription start site in prostate tumors, that we had analyzed by MeDIP-Seq [22], and found this region to be differentially methylated between tumors with and without the *TMPRSS2:ERG* gene fusion. Specifically, the 1 kb region spanning the *TDRD1* promoter-associated CpG island was significantly hypomethylated in the *TMPRSS2:ERG*-positive tumors compared to benign and *TMPRSS2:ERG*-negative tumors (P<0.0001, Fig. 3a). A 500-bp window immediately downstream of the CpG island did not show differences in DNA methylation between ERG-negative and ERG-positive tumors (P = 0.41, Fig. 3a). Moreover, DNA sequence flanking the putative *TDRD1* promoter was not differentially methylated between any of the three groups (P>0.05), indicating that the differential DNA methylation occurs in the direct proximity of the *TDRD1* transcriptional start site but not around it. Of note, the average level of *TDRD1* promoter methylation was inversely correlated with *TDRD1* mRNA levels across all 93 samples (*ρ* = -0.57), suggesting that loss of promoter methylation substantially contributes to *TDRD1* overexpression in *TMPRSS2:ERG* fusion-positive prostate cancer (Fig. 3b).

### ERG-induced Loss of Epigenetic Repression at the *TDRD1* Promoter is a Major Mechanism of *TDRD1* Activation

**Figure 4:**
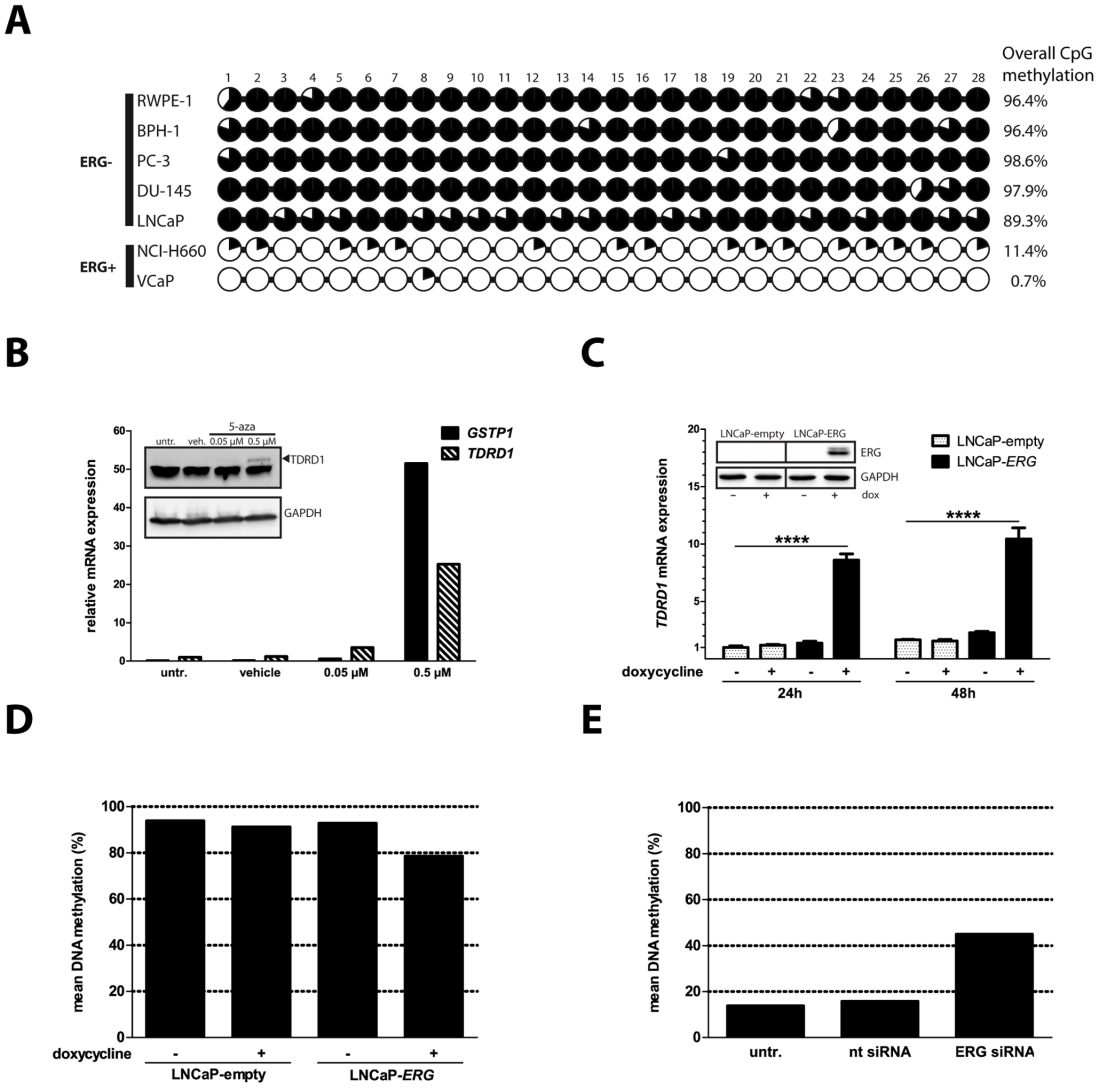
ERG-induced loss of epigenetic repression at the *TDRD1* promoter is a major mechanism of *TDRD1* overexpression. (A) DNA methylation analysis of the *TDRD1* promoter-associated CpG island in prostate cell lines by bisulfite sequencing. Average methylation level of the whole CpG island calculated from five sequenced colonies is shown (%). (B) Analysis of mRNA expression in LNCaP cells after treatment with the demethylating agent 5-aza-2′-deoxycytidine. Insert: analysis of protein expression in LNCaP cells after treatment with 5-aza-2′-deoxycytidine. 75µg of protein lysate from LNCaP cells was used per lane. (C) Analysis of mRNA expression in stable LNCaP clones overexpressing *ERG*. Two independent experiments were performed in triplicate. Insert: ERG expression analysis at 48 h in LNCaP clones by western blotting. (D) Bisulfite sequencing of the *TDRD1* promoter-associated CpG island in LNCaP cells 48 h after induction of ERG expression with doxycycline. (E) Bisulfite sequencing of the *TDRD1* promoter-associated CpG island 96 h after silencing of *ERG* in VCaP cells. The data shown in (D) and (E) are mean % of methylation of the entire CpG island calculated from 11–12 sequenced clones.

Since the differentially methylated region spanned the CpG island associated with the *TDRD1* promoter and CpG islands are known to play a role in regulating transcription [49], we have performed bisulfite sequencing of the *TDRD1*-associated CpG island in prostate cell lines. We found that the CpG island was fully methylated in benign cells and ERG-negative cancer cell lines, with an average methylation ranging from 89.3% for LNCaP to 98.6% for PC-3 (Fig. 4a). In contrast, CpG methylation was almost completely absent in the *TMPRSS2:ERG*-positive cell lines NCI-H660 and VCaP (11.4% and 0.7%, respectively). Comparison of *TDRD1* mRNA levels (Fig. 1b) to the DNA methylation of the CpG-island at the *TDRD1* promoter (Fig. 4a) revealed an inverse correlation between the two parameters across the investigated cell lines, which was in accordance with the corresponding data from prostate tumors. Given the stark differences in DNA methylation at the *TDRD1* promoter between the ERG-rearranged and remaining cell lines, we hypothesized that loss of DNA methylation at the *TDRD1* promoter is the major mechanism responsible for *TDRD1* activation. To test this possibility, we treated ERG-negative LNCaP cells with the DNA methyltransferase inhibitor 5-aza-deoxycytidine (decitabine; [50]). After five days of treatment with submicromolar concentrations of decitabine we observed a dose-dependent increase in expression of *GSTP1* gene which is known to be silenced by methylation in LNCaP cells [51] (Fig. 4b). Notably, *TDRD1* mRNA was upregulated by more than 25-fold. Consequently, following the increase in *TDRD1* mRNA levels, TDRD1 protein became detectable in LNCaP cells by immunoblotting (Fig. 4b, insert), Indicating that loss of DNA methylation may indeed be sufficient to drive *TDRD1* expression.

To check if ERG can mimic the effects exerted on *TDRD1* expression by demethylation of the LNCaP genome, we generated stable LNCaP cells overexpressing coding sequence of the *TMPRSS2:ERG* fusion T1/E4 in an inducible manner. Induction of *ERG* expression with doxycycline led to an almost 5-fold increase in *TDRD1* mRNA, while doxycycline had no influence on *TDRD1* expression in the LNCaP clone carrying the empty expression vector (Fig. 4c). We have used bisulfite sequencing to analyze the corresponding DNA methylation status of the *TDRD1* promoter-associated CpG island upon ERG induction. ERG overexpression led to the hypomethylation of the *TDRD1* promoter region in 27% of the investigated alleles, while we did not observe any hypomethylation upon doxycycline treatment in the empty vector control (Fig. 4d, Fig. S1a). Given that the converse experiment, i.e. depletion of ERG in VCaP cells by RNAi, has led to downregulation of TDRD1 expression (Fig. 2) we have also performed bisulfite sequencing of the *TDRD1* CpG island after ERG silencing in VCaP cells. At 96 h post-transfection, silencing of ERG with 65% efficiency resulted in almost 3-fold increase in mean DNA methylation at the CpG island, from 15.7% of methylated CpGs in non-targeting control to 45% in cells treated with siRNA targeting *ERG* (Fig. 4e, Fig. S1b).

The above mentioned observations demonstrate that DNA methylation status of the *TDRD1* promoter and thus its transcriptional activity are mechanistically linked to the levels of ERG transcription factor in prostate cancer cells.

### Differential Role of *TDRD1* in Testis and Prostate Cancer

**Figure 5:**
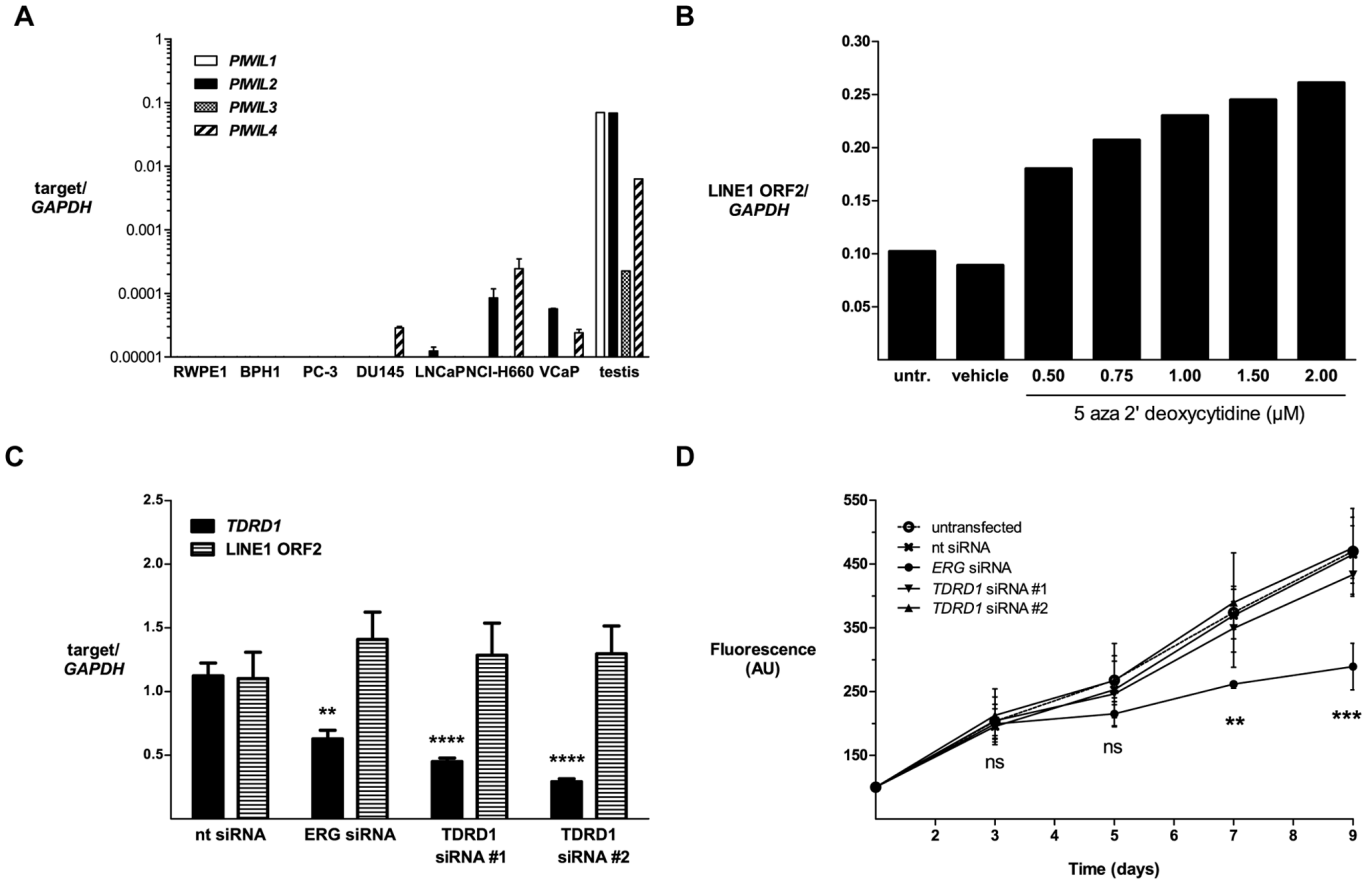
*TDRD1* does not control LINE1 activity in *TMPRSS2:ERG*-positive VCaP cells. (A) mRNA expression analysis of *PIWIL* genes in prostate cell lines by qRT-PCR. Testis RNA was used as a positive control. (B) mRNA expression analysis of LINE1 ORF2 in VCaP cells following 5 days of treatment with 5-aza-2′-deoxycytidine. (C) mRNA expression analysis of LINE1 ORF2 in VCaP cells following prolonged (8 days) *ERG* or *TDRD1* silencing. (D) Metabolic viability assay of VCaP cells treated with siRNAs. One (B), two (A) or three (C, D) independent experiments were performed in triplicate.

The evolutionary conserved piRNA pathway plays an important role in male germline during spermatogenesis [52]–[54]. The components of the piRNA pathway act to suppress the activity of transposable elements, potentially in order to maintain the integrity of the germline chromosomes during genome-wide demethylation in primordial germ cells [55]–[57]. Studies performed in mouse and zebrafish have shown that Tdrd1, ortholog of human *TDRD1*, is a component of the piRNA pathway which specifically interacts with piRNA-associated proteins to potentiate the piRNA-mediated silencing of LINE1 retrotransposons. Accordingly, loss of Tdrd1 in mouse was shown to result in LINE1 derepression [27], [58]. In humans, four orthologs coding for piRNA-associated proteins exist: *PIWIL1*-*PIWIL4* [59]. To test if TDRD1 may interact with PIWI proteins in *TMPRSS2:ERG*-positive prostate cancer and thus contribute to the piRNA pathway activity, we measured the mRNA expression of human PIWIL genes in prostate cancer cell lines (Fig. 5a). mRNA expression of *PIWIL1*-*PIWIL4* genes was undetectable by qRT-PCR in most of the prostate cell lines investigated. In cell lines with a detectable expression, *PIWIL* mRNA levels were >500-fold lower than in testis, which was used as a positive control, thus making it unlikely that the piRNA pathway is functional in prostate cancer cells. Despite hardly detectable *PIWIL1-4* expression, we decided to test the extent of TDRD1 influence on LINE1 retrotransposition activity in prostate cancer cells. We have measured mRNA expression of the LINE1-encoded endonuclease (L1 ORF2), which we used as an approximation for LINE1 activity, after TDRD1 depletion in VCaP cells. As a positive control for our assay, we observed a dose-dependent induction of L1 ORF2 upon treatment of LNCaP cells with increasing concentrations of the demethylating agent decitabine (Fig. 5b). However, even after prolonged (8 days) and effective *TDRD1* silencing in VCaP cells we observed no significant differences in LINE1 expression (Fig. 5c), indicating that TDRD1 abundance does not control LINE1 activity in *TMPRSS2:ERG*-positive prostate cancer cells. Moreover, in contrast to silencing of ERG, which is known to negatively influence *in vitro* growth of VCaP cells, silencing of *TDRD1* did not have any impact on VCaP cell viability (Fig. 5d). Accordingly, overexpression of TDRD1 in ERG-negative LNCaP cells did not lead to changes in cell viability (data not shown), suggesting that expression of *TDRD1* is not required for proliferation of ERG-rearranged prostate cancer cells *in vitro*.

## Discussion

Previous studies have identified *TDRD1* as the most differentially expressed gene between *ERG*-negative and *ERG*-positive prostate tumors besides *ERG* [16], [23], [60], [61]. Herein, we describe the co-expression of *ERG* and *TDRD1* in prostate cancer *in vitro* and *in *vivo. We demonstrate that *TDRD1* expression is induced by the ERG transcription factor in *TMPRSS2:ERG-*rearranged prostate cells. We show that the *TDRD1* promoter is hypomethylated in *TMPRSS2:ERG-*rearranged tumors and cell lines and report that the DNA methylation inversely correlates with *TDRD1* expression *in vivo*. In these regards, our data extend and corroborate the findings of a recently published study [61]. In addition, we functionally link ERG rearrangements to *TDRD1* overexpression by presenting mechanistic evidence that it is the accumulation of ERG which leads to loss of the DNA methylation at the *TDRD1* promoter. Thus, we propose the existence of ERG-induced epigenetic activation of gene expression.

Our data suggest that activation of *TDRD1* transcription is a consequence of *ERG* but not of *ETV1* rearrangements. This is in agreement with the data by Paulo et al., who experimentally classified genes differentially expressed in fusion-positive primary prostate tumors into three distinct categories: ERG-targets, ETV1-targets and overlapping targets and showed that *TDRD1* belongs to the first category [61]. Similarly to Paulo et al., we have also found an inverse correlation between *TDRD1* expression and DNA methylation of the *TDRD1* promoter *in vitro* and *in vivo*. A restricted tissue expression pattern of *TDRD1* (expressed in the germline and silent in adult somatic tissues [26], [62]) is likely governed by extensive methylation of the *TDRD1* promoter-associated CpG island [26]. This view is further supported by a repression of *TDRD1* expression accompanied by complete CpG methylation in benign prostate tissues and fusion-negative prostate cancer. Our data shows that it is either ERG or factors acting downstream of ERG which are responsible for the loss of DNA methylation at the *TDRD1* promoter observed in *TMPRSS2:ERG*-positive tumors. We present two independent experimental proofs of the statement above: i) forced expression of ERG in LNCaP cells is sufficient to activate *TDRD1* expression and is accompanied by a loss of DNA methylation at the *TDRD1* promoter, ii) *ERG* silencing in VCaP cells is sufficient to restore the tissue-specific methylation status at the *TDRD1* promoter and is accompanied by a repression of *TDRD1* transcription. Given that ERG was shown to bind DNA upstream of the *TDRD1* transcription start site [61] and that transcription factor binding was suggested to exert a protective role against CpG island methylation [48], [63], we propose a model in which ERG binding leads to loss of DNA methylation at the *TDRD1* promoter. This could be accomplished by two alternative modes of action: active, in which ERG recruits enzymatic activities to remove DNA methylation or passive, in which ERG competes with DNA methyltransferases for their binding sites in the proximity of *TDRD1* promoter, thereby preventing maintenance of DNA methylation during DNA replication. In this context, it is interesting to note that a recently published study reported *TDRD1* promoter to be hypermethylated in infertile male patients with spermatogenic disorders [64], linking *TDRD1* promoter methylation and *TDRD1* expression to human disease.

Close inspection of data reveals that in several prostate tumors tested negative for the *TMPRSS2:ERG* rearrangement, *TDRD1* is expressed at high levels despite low *ERG* expression, suggesting that ERG may not be the only factor which is capable of activating *TDRD1* transcription. Such *ERG^low^*/*TDRD1* ^high^ tumors were also reported by Taylor et al. [17]. ERG-independent induction of *TDRD1* could also explain the apparently counter intuitive observation that in NCI-H660 cells the *TDRD1* promoter is hypomethylated, despite the absence of detectable ERG protein. Thus, the possibility of other factors controlling *TDRD1* expression in prostate cancer cells cannot be excluded.

One of the important questions brought up by this study concerns the consequences of TDRD1 accumulation in ERG-rearranged prostate cancer. Our findings suggest that TDRD1 does not contribute to the control of LINE1 activity in prostate cancer cells, as it is unlikely that the piRNA pathway is functional these cells. Despite the fact that TDRD1-interacting proteins have not been identified in humans, a human ortholog (*PIWIL2*) of a mouse gene coding for a Tdrd1-interacting protein (Mili) is either not-expressed or expressed at very low levels in prostate cell lines. Furthermore, silencing of *TDRD1* or *ERG* did not influence the activity of LINE1 elements. In addition, we did not observe TDRD1 to be essential for viability and proliferation of prostate cancer cells *in vitro.* While it is conceivable that *TDRD1* expression confers a selective advantage to prostate cancer cells only *in vivo*, we cannot exclude that the impact of *TDRD1* silencing on cell viability *in vitro* may be masked by the expression of another protein with a redundant function. Irrespective of the possible functional involvement of *TDRD1* in prostate cancer, TDRD1 overexpression has a potential of being exploited in prostate cancer therapy. It is well established that ERG expression is not entirely specific to ERG-rearranged prostate cancer cells: ERG is known to be expressed in endothelial cells and in the hematopoietic lineage. In contrast, as a cancer/testis antigen, *TDRD1* is not expressed in healthy somatic tissues. *TDRD1* expression in testicular tissue does not constitute a potential risk of the off-target activity for immunotherapy due to the immunological privilege of testis. We thus propose that overexpression of *TDRD1,* observed by us and others in 100% of *TMPRSS2:ERG*-positive prostate tumors, makes *TDRD1* a promising target for immunotherapy of ERG rearrangement-positive prostate cancer.

In conclusion, we report here that overexpression of ERG transcription factor in *TMPRSS2:ERG*-positive prostate cancer induces a loss of DNA methylation at the *TDRD1* promoter-associated CpG island. By providing the evidence of a mechanistic link between ERG and methylation we uncover a previously undescribed phenomenon of ERG-induced epigenetic gene activation. Finally, our data suggest that *TDRD1* overexpression in *ERG*-rearranged prostate cancer has a potential of being exploited as a target for prostate cancer immunotherapy.

## Supporting Information

Figure S1 **Levels of ERG modulate DNA methylation at the** ***TDRD1*** **promoter in prostate cancer cells.** Bisulfite sequencing of the *TDRD1* promoter-associated CpG island (A) 48 h after induction of forced *ERG* expression in LNCaP cells or (B) 96 h after knockdown of *ERG* by RNAi in VCaP cells. Each circle represents a single CpG dinucleotide of the CpG island (black: methylated, white: unmethylated).
(TIF)

Table S1 (XLS)
