# Supplementary figures and images for: ERG Induces Epigenetic Activation of Tudor Domain-Containing Protein 1 (TDRD1) in ERG Rearrangement-Positive Prostate Cancer
Source: PLoS One. 2013 Mar 29;8(3):e59976. doi: 10.1371/journal.pone.0059976 (PMC3612037; doi:10.1371/journal.pone.0059976)

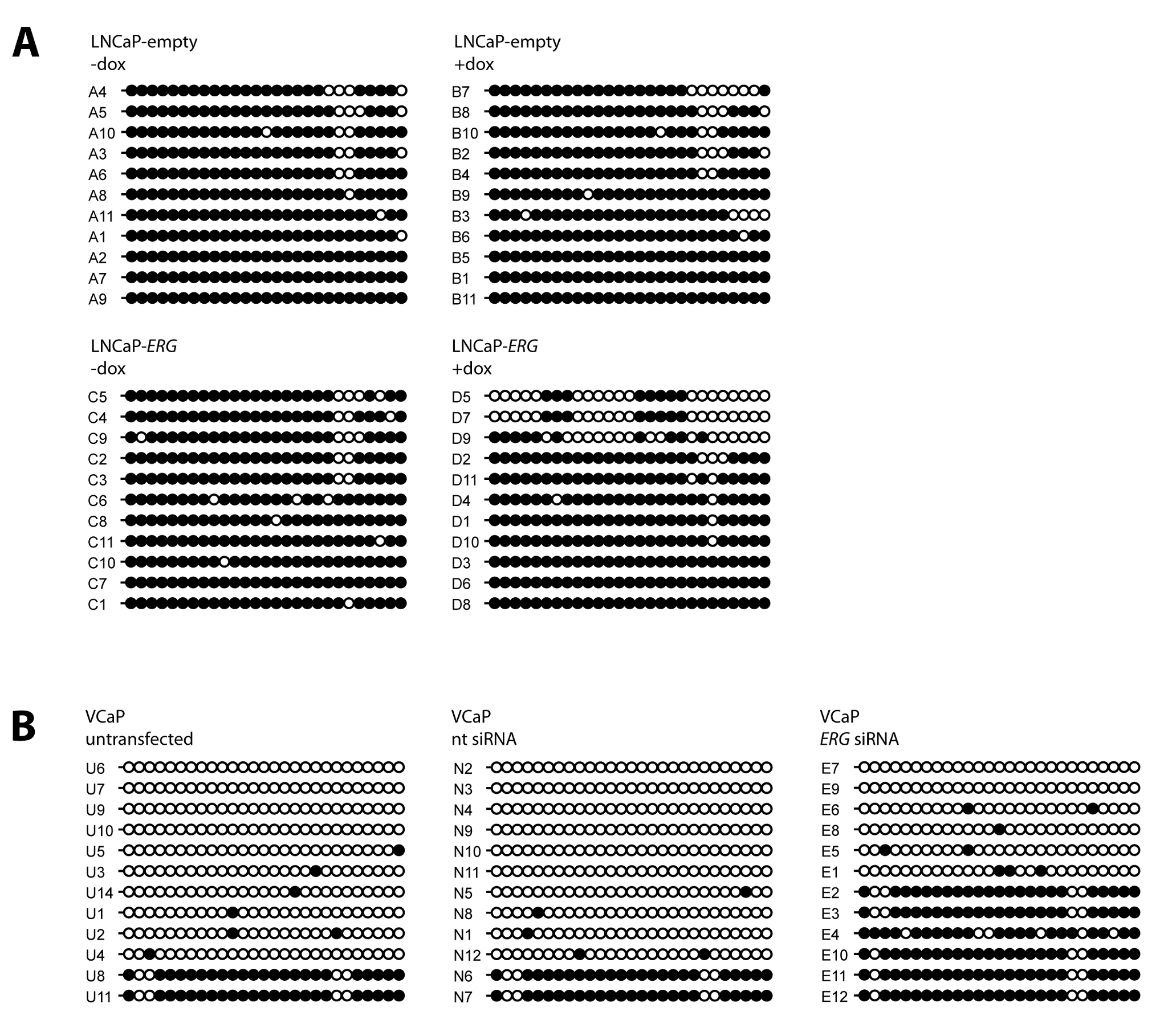

Supplement: Figure S1 — Levels of ERG modulate DNA methylation at the TDRD1 promoter in prostate cancer cells. Bisulfite sequencing of the TDRD1 promoter-associated CpG island (A) 48 h after induction of forced ERG expression in LNCaP cells or (B) 96 h after knockdown of ERG by RNAi in VCaP cells. Each circle represents a single CpG dinucleotide of the CpG island (black: methylated, white: unmethylated). (TIF) [file pone.0059976.s001.tif]
